# The Future of Radiology

**DOI:** 10.5041/RMMJ.10059

**Published:** 2011-07-31

**Authors:** Alexander R. Margulis

**Affiliations:** Professor of Radiology (Emeritus), Department of Radiology, New York Presbyterian Hospital, Weill Cornell Medical Center, New York, USA

It has been my good fortune to live and practice radiology during a long period of momentous change – to see the transformation of the discipline from a supportive service into a mainstream, essential branch of clinical medicine. I remember wearing red goggles to adapt my vision before performing fluoroscopy; observing the horrible, now thankfully obsolete, practice of ventriculography, which was considered advanced neuroradiology; and performing other, now rarely prescribed procedures, such as double-contrast barium enemas and intravenous pyelography. Witnessing the beginnings of interventional radiology, I suggested its name in an editorial.^1^ I also had the good fortune to see the introduction of computed tomography (CT) and a technology first known as nuclear magnetic resonance imaging. Together with fellow members of a committee of the American College of Radiology and editors of prestigious radiological journals, I took part in changing the name of the latter modality to MRI, freeing it from threatening implications.

Looking back on these experiences, one lesson stands out above all: Innovation and transformation never cease. Looking forward, it is clear that radiology, along with the rest of medicine, is now undergoing further momentous changes that will affect the future of all those already practicing as well as those yet to start their careers.^2^

## TRENDS IN MEDICINE AND RADIOLOGY: SUB-SPECIALIZATION AND TECHNOLOGICAL ADVANCEMENT

Considering historical trends in medicine may allow us to anticipate better the future. In the course of the last century, total knowledge in medicine grew not linearly but exponentially, and as a result everyone in medicine had to know more and more about less and less. Radiology followed the path taken by the rest of medicine and sub-specialized in order to remain a valuable contributor. Increasingly specialized clinicians expected answers to questions that would affect treatment, and so radiologists needed to know specialty fields in detail. These practical demands required that radiology training programs adapt, and academic departments were soon peopled with faculty members who taught and performed research in sub-specialties.

At the same time, radiology benefited from continual advances in imaging technology and, in the last thirty-five years, cybernetics. Together, technological prowess and specialization have enabled radiology to become a crucial participant in every aspect of modern medical practice, from diagnosis to treatment selection and follow-up, and even treatment itself. Looking ahead, we can expect not only further sub-specialization and technological advancement, but also even deeper integration of radiology into all facets of medicine.

## FUTURE DEVELOPMENTS IN DIAGNOSTIC RADIOLOGY

Like the rest of medicine, radiology will need to become even more multi-directional as it remains in step with contemporary scientific and technological advances. In the immediate future, this will mean more applications of hybrid imaging technology such as PET/CT as well as incorporation of sonography in many procedures, including interventional procedures performed with robotics.

Over the longer term, radiology, along with all other medical disciplines, will move massively into the molecular age using all its available technologies. It will become predictive and rapidly personalized. We are already seeing the beginnings of this shift – for example, in the application of hybrid imaging and the development of imaging biomarkers predictive of early response.^3^ The use of genetic biomarkers, while still in its infancy, has already changed approaches to treatment for some diseases, such as cystic fibrosis.^4^

In the next decade and beyond, imaging will evolve at a still more rapid pace, as new knowledge is acquired about metabolic processes, and, in turn, tracers are developed that identify the mechanisms of metabolic processes, further enhancing our ability both to acquire and apply new knowledge. Such tracers will be created not only for PET/CT but for the emerging technology of MR/PET, which can combine the advantages of freshly developed tracers with the benefits of innumerable MR imaging sequences as well as MR spectroscopy.^4^ Integrating molecular imaging with genetic markers promises to offer additional, powerful approaches to personalizing medicine.^5^

## FUTURE DEVELOPMENTS IN INTERVENTIONAL RADIOLOGY

Interventional radiology is already in a state of flux, employing all the presently available imaging modalities for guidance, combining them for greater precision, and applying them with multiple treatment technologies (e.g. thermal, radiofrequency, and laser ablation tools).^6^ Remarkably precise guidance of finer and finer catheters and the addition of molecular and genetic markers will transform body and neuro-interventional radiology from a service limited to correcting vascular abnormalities to a major therapeutic tool in body and cerebral oncology, offering highly localized ablation with injection of targeted therapeutic agents. The introduction of genetic markers into radiology will expand the use of image-guided interventions.^7^

## EDUCATION AND TRAINING OF FUTURE RADIOLOGISTS

The future of radiology is more than promising. It will greatly depend, however, on our ability to change the education and training of incoming young physicians and involve them heavily in the emerging era of molecular imaging and molecular medicine. A period of obligatory research should be introduced into the training programs, as our discipline is undergoing a major transition, and participating in its advancement is both educational and inspirational. Such requirements already exist in training programs in surgery and internal medicine.

## CAVEAT

To conclude, much of our well-being, and the advancement of all disciplines, depends on economic stability and the absence of wars. Hopefully we shall continue to live in our apparently fragile peace, our recessions will be mild, and science, including medicine, will be allowed to progress and prosper. While technologies may advance or even (as in the case of sonography) be created in wartime, science requires peace and the dedication and energy of a civilian society.
